# Isotopic study of intraseasonal variations of plant transpiration: an alternative means to characterise the dry phases of monsoon

**DOI:** 10.1038/s41598-018-26965-6

**Published:** 2018-06-05

**Authors:** S. Chakraborty, A. R. Belekar, A. Datye, N. Sinha

**Affiliations:** 1 Indian Institute of Tropical Meteorology, Pune, India; 2 Department of Atmospheric and Space Sciences, Savitribai Phule Pune University, Pune, India; 3 Department of Environmental Science, Savitribai Phule Pune University, Pune, India

## Abstract

The isotopic characteristics of plant transpired water are strongly controlled by soil evaporation process, primarily by relative humidity. The monsoon system is characterised by large variability of several atmospheric parameters; the primary one being the rainfall, which in turn, modulates the relative humidity. Due to the strong dependency of transpiration on relative humidity, it is expected that this process would vary in accordance with the active and break periods of the monsoon season, which are known to produce cycles of humid and relatively dry phases during a monsoon season. To study the transpiration process, an experiment was conducted wherein rainwater and transpired water were collected from a few plants and analyzed for their isotopic ratios during the summer monsoon seasons of 2016 and 2017. The difference between the isotopic characteristics of the transpired water and rain water is expected to be nominally positive, however, a large variability was observed. This difference is found to be high (low) during the reduced (enhanced) humidity conditions and varies in tandem with the break and active phases of the monsoon season. This characteristic feature may thus be used to delineate the dry and wet phases of monsoon on local to regional scale.

## Introduction

Plant transpiration comprises a significant source of water vapour into the atmosphere. Trees and plants absorb a large amount of soil water to carry out their physiological processes, which is eventually released into the atmosphere in the form of transpiration. Hence, for a better understanding of the hydrological system, the study of transpiration or more generally, the evapotranspiration (ET) is undertaken by the hydrologists, the plant and agricultural scientists. It is believed that ET plays an important role in the global circulation of the atmosphere and the precipitation processes^1^. It is estimated that ET could generate up to 50% of the total moisture for precipitation in tropical rainforests and therefore acts as a crucial component in regional moisture budget^2–4^. Considering the recent changes in global precipitation pattern^5^, monitoring the long-term changes of ET has gained considerable importance^6,7^. However, precise estimation of evapo-transpired moisture, especially that due to the plant transpiration processes and knowledge of its local variability remains poorly constrained due to scanty observational network^8^. The existing methods that are commonly used for estimating the transpiration fluxes are individual-tree sap flux^9^, whole tree chamber observations^10^ and paired soil lysimeters^11^. Additionally, the isotope-based measurements seem to hold promise in this endeavour^12^. The isotopic composition of transpiration (δ^18^O_Tr_) is a complex function of several environmental parameters. The primary factors include the isotopic composition of the liquid water at the evaporation site, the local atmospheric water vapour and the relative humidity in the ambient air. Several other internal and external environmental conditions of the leaf are believed to affect the isotopic properties of the transpired water^13–15^.

The determination of the isotopic composition of transpiration usually relies on indirect methods, such as stem water measurements, leaf water measurements^16^ or process-based modeling^17^. The first direct measurements of δ^18^O_Tr_ were reported by Harwood *et al*.^18^ in 1998, who used a cold trap technique to collect transpired vapours and measured them using an isotope ratio mass spectrometer. Subsequently, a technique to measure the isotopic composition of the transpired vapour in real time using a laser-based isotope analyzer was developed^19^. The same technique was modified and implemented for the real-time field observations^20^. On the other hand, a simplified off-line collection method was proposed^21^ whereby a traditional method of plastic bag or gas chamber was used to collect transpired water integrated for 24 hours followed by mass spectrometric analysis. As mentioned earlier, the transpiration process, is largely controlled by a few atmospheric variables. While temperature (T) and wind speed (W) affect it in a positive manner, the relative humidity (RH), imparts a negative effect on this process^22^. Additionally, plant parameters, such as stomatal conductance, leaf cuticular thickness, etc., also play some role, but to a much lesser extent relative to the atmospheric parameters.

Indian summer monsoon brings copious rain to the subcontinent during the summer season spanning typically from June to September. Most of the moisture during this time is derived from the neighbouring oceans. But a significant amount of continentally derived moisture is believed to be generated as well, especially during the dry phases of the monsoon when the rainfall is less and the humidity is low; however a prolonged dry phase could deplete the soil moisture content resulting in shifting of the available surface energy from the latent to the sensible heat^23^. The mechanism of leaf water enrichment process is reasonably well understood and fractionation models have been proposed by some authors^24^, for example, to predict the oxygen isotopic composition of plant organic matter^25^ aimed at paleoclimatic reconstruction^26,27^. In this work, we investigate the isotopic behaviour of transpired water that inherits its isotopic signature from the leaf water, which is subsequently modulated by the atmospheric variables, especially due to the changes in humidity and other environmental parameters during the monsoon season. Reduced humidity is known to enhance the evaporation and transpiration processes^22^, but the consequent changes in isotopic composition of transpired water/vapour in response to monsoon system, to our knowledge, has not yet been studied. A pertinent question is- what is the nature of the isotopic variability of the transpired water during the wet and dry phases of the monsoon season?

To address this issue, we have done a controlled experiment wherein transpired water was collected from four potted plants placed in a small canopy during the monsoon season of 2016 and analysed for isotopic variations. To provide more credence, the same experiment was continued during the 2017 monsoon season on the natural plants. The main objectives of the current study are to figure out how the isotopic composition of plant transpiration is affected by the monsoonal variation on the sub-seasonal time scale, and in turn, if that behaviour could be used to study the hydro-meteorological processes.

## Study Area

An urban environment within the premises of the Indian Institute of Tropical Meteorology (IITM), Pune was chosen for this study. The area is covered by adequate vegetation consisting of deciduous trees and understory plants with a total average canopy height of 20 m. Rainwater was also collected from this site as well as from Dhankawadi, a site situated approximately 14 km south of the IITM campus. Details are provided in the Method section.

## Results

**Figure 1:**
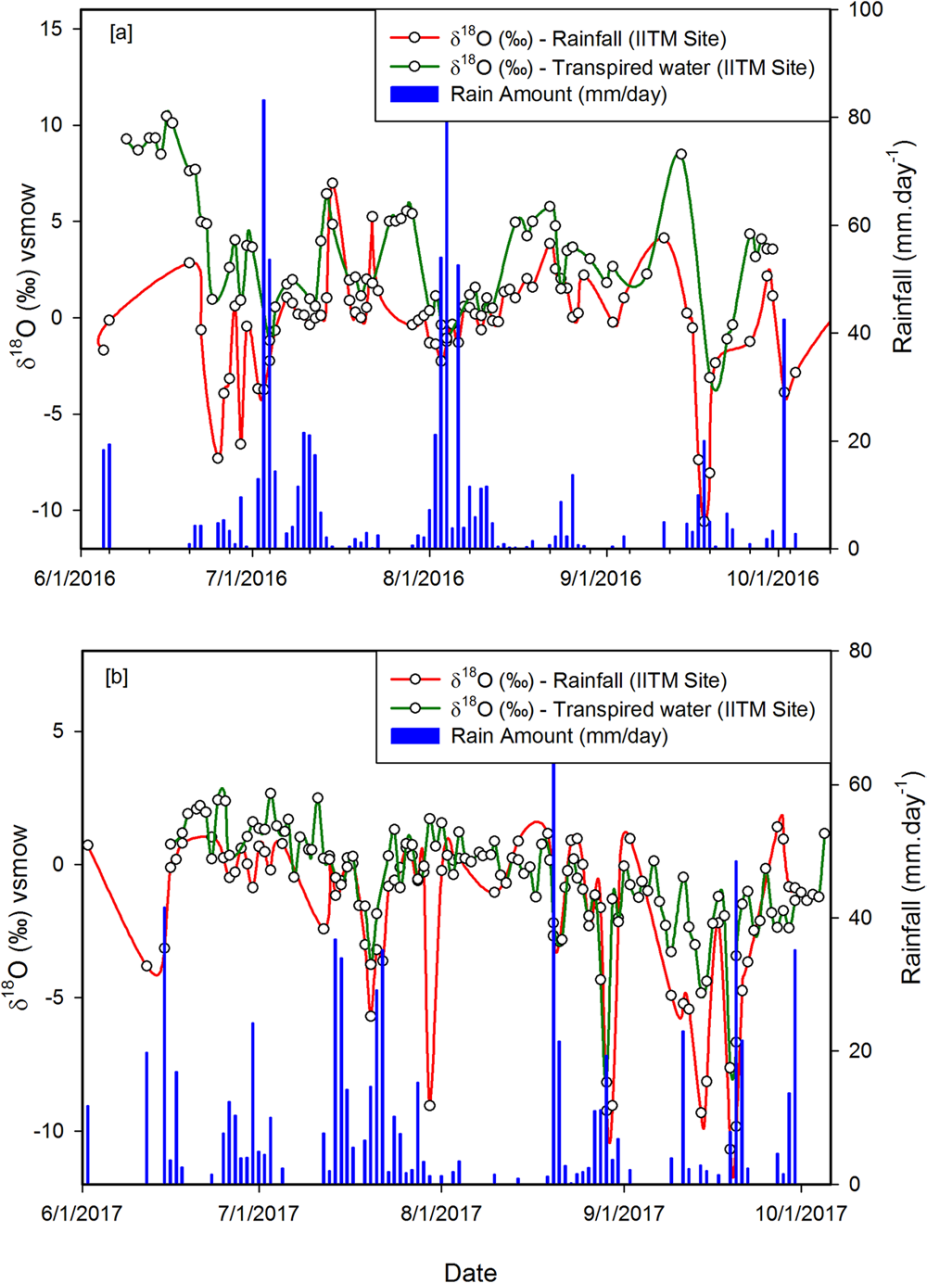
Isotopic time series of rainfall and transpired water. (**a**) Rainfall and its isotopic values; Site: IITM. The blue bars show the rainfall. The red line indicates the rainwater isotopic variability. The green line indicates the transpired water isotopic variability. (**b**) Same as above, but for the year 2017. The plot was made using a licensed copy of SigmaPlot.

Figure 1a shows the rainfall time series (bars). The isotopic values are shown as lines. The red line indicates the isotopic values of rainfall (δ^18^O_Rn_) for the IITM site, while the green line represents the isotopic values (average) of the four plants studied during the year 2016. Figure 1b illustrates the same, but for the year 2017. The rainfall and its oxygen isotopic time series for the Dhankawadi site are presented in Fig. S1.

**Figure 2:**
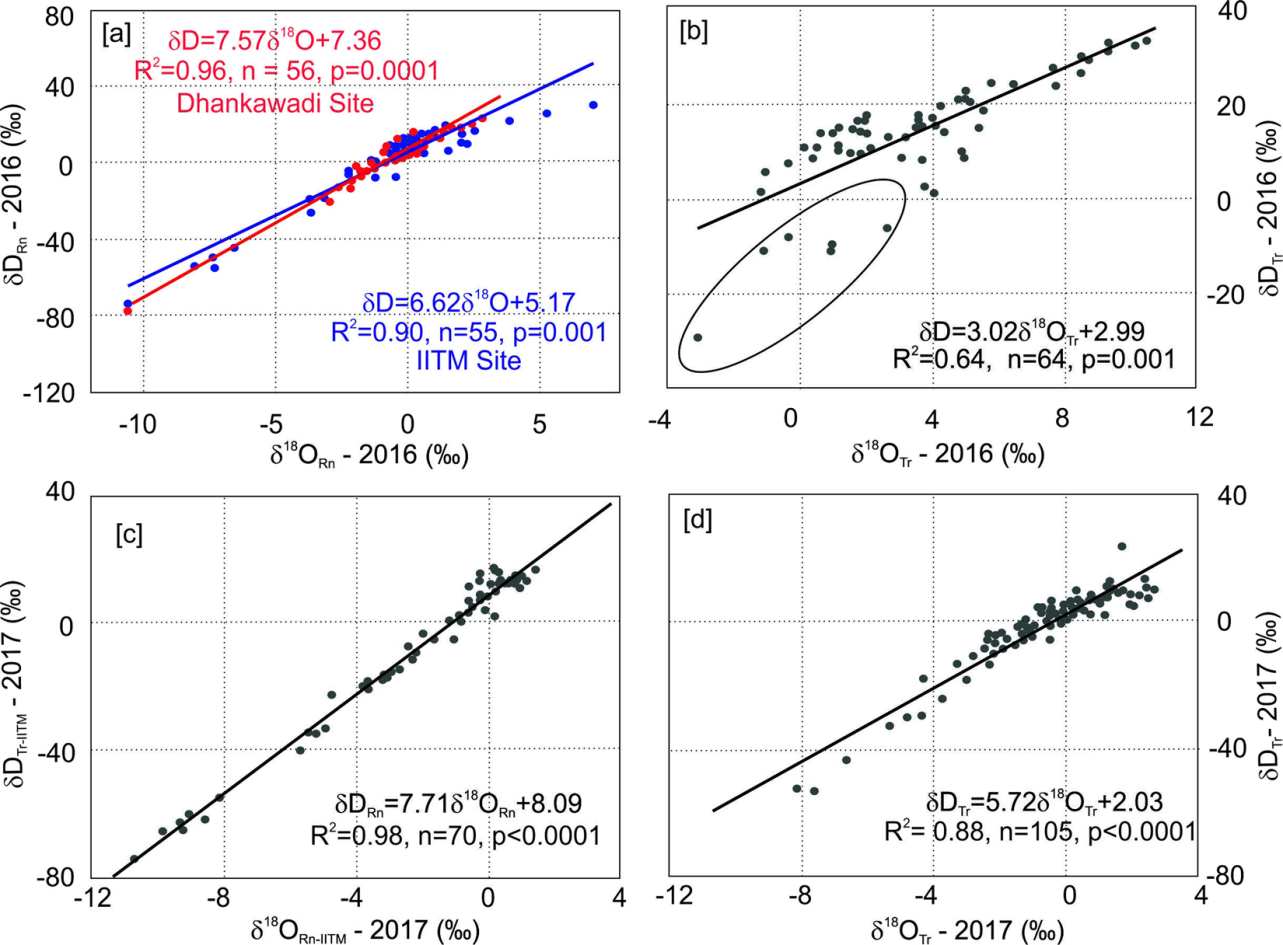
Meteoric water and evaporation Line. (**a**) The local meteoric water lines for the IITM site (blue line) and the Dhanakwadi site (red line). (**b**) The evaporation line in the transpired water (Plant-1) is shown here. (**c**) The local meteoric water line for the IITM site for the year 2017 and (**d**) The evaporation line for the transpired water of the natural plant for the year 2017. The plot was made using a licensed copy of SigmaPlot.

The local meteoric water lines (LMWL) for both the sites have been shown in Fig. 2a for 2016. The IITM-LMWL is shown in blue and the Dhankawadi-LMWL is shown in red. The slope (intercept) for these sites are 6.62 (5.17) and 7.57 (7.36) respectively. The δ^18^O − δD plot for the transpired water (denoted as ‘evaporation line’) is shown in Fig. 2b (Plant-1); the slope and intercept are 3.02 and 2.99 respectively. Some points (encircled) in this case appear to be distinctly different from the rest of the points. Possible reasons for such anomaly and its implications will be discussed later. The LMWL for the year 2017 has been shown in Fig. 2c (slope = 7.71, intercept = 8.09). Figure 2d represents the relationship between δ^18^O and δD for the transpired water for the year 2017. The slope of the transpired water line for the natural plant (5.72) is significantly higher than that for the potted plant.

## Discussion

The local meteoric water lines at the IITM and the Dhankawadi sites reveal somewhat different behaviour. For example, the slope (6.62) and intercept (5.17) of the IITM site are lower than that of the Dhankawadi site (7.57 and 7.36 respectively). A slope lower than 8 (the slope of the Global Meteoric Water Line: GMWL) usually means the raindrops are undergoing kinetic fractionation, i.e., evaporative enrichment when raindrops are descending through the atmosphere below the cloud base. In case of Dhankawadi, the slope (7.57) is not significantly different than the GMWL slope of 8. This indicates that raindrop at this place did not suffer much evaporation. On the other hand, the slope at the IITM site (6.62) is considerably lower than 8 implying significant amounts of the raindrops are evaporated during the rainout process in this area. This is also corroborated by the δ^18^O vs. d-excess (defined as d_Rn_ = δD_Rn_ − 8*δ^18^O_Rn_ for rain water) correlation, since raindrops evaporation leads to an inverse correlation between these two parameters^28^.

**Figure 3:**
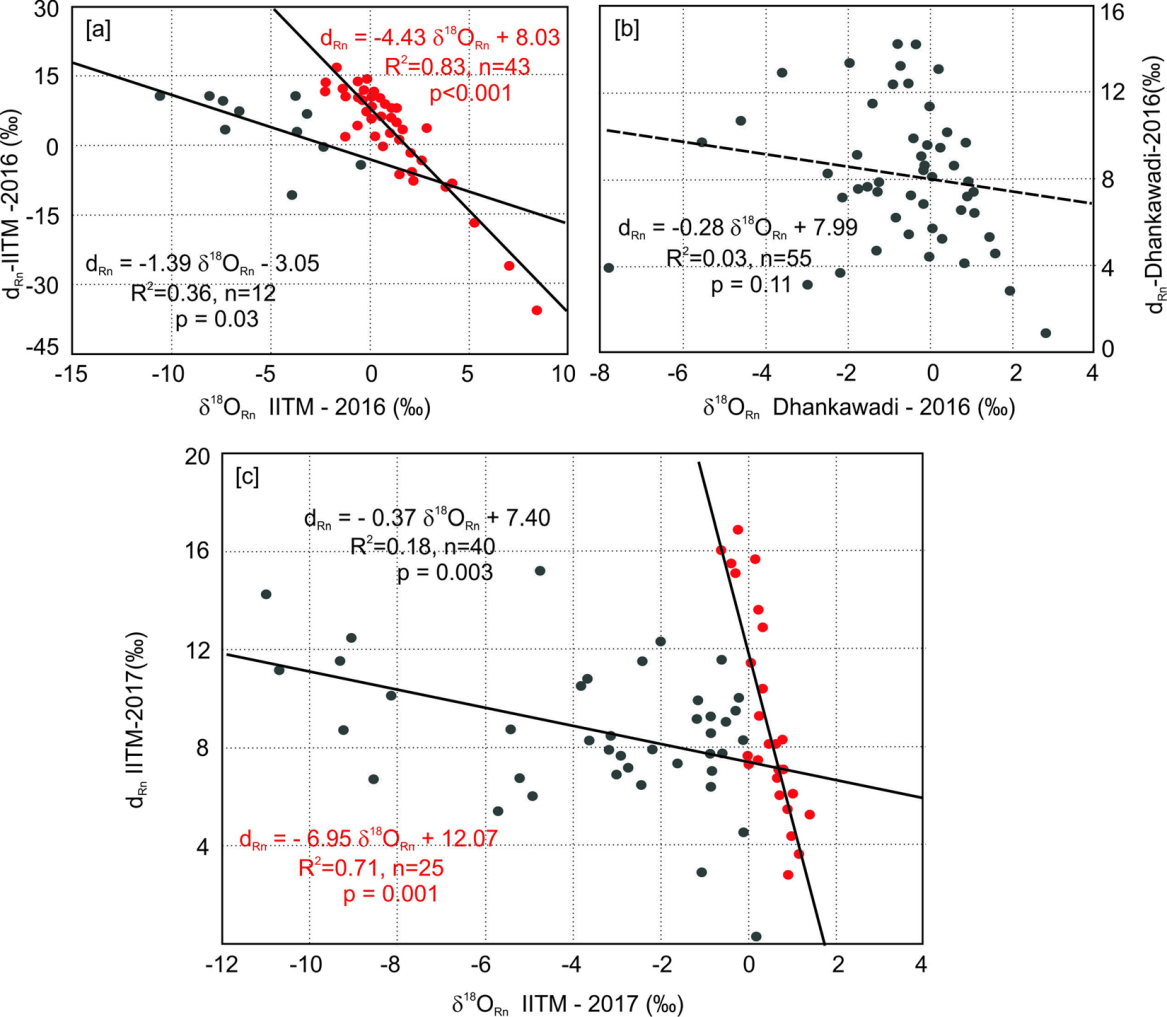
Relation between δ^18^O and d-excess of rainfall: (**a**) IITM 2016, (**b**) Dhankawadi 2016, (**c**) IITM 2017. Red circles characterise stronger raindrop evaporation represented by higher slopes. The plot was made using licensed a copy of SigmaPlot.

The δ^18^O_Rn_ vs. d_Rn_ relationship of the IITM site is shown in Fig. 3a and demonstrates two distinct trends. The first set consists of data points (black) that are characterised by a lower slope (−1.39). The second set consists of data points (red) with a much steeper slope (−4.43). This implies that the second set of data points represented a system that underwent stronger evaporation than that observed in the first dataset. On the other hand, the distribution of the data points in the δ^18^O_Rn_ vs. d_Rn_ plot for the Dhanakwadi site yields a very weak correlation (R^2^ = 0.03, Fig. 3b). This is a strong indication that the rainfall in this area did not suffer significant evaporation. These characteristic features of these two sites imply that the extent of recycled moisture is higher at the IITM site compared to the Dhankawadi site. However, the situation was somewhat different for the year 2017. The extent of raindrop evaporation, for the overall season at the IITM site is less compared to the year 2016, as these parameters (δ^18^O_Rn_ and d_Rn_) when regressed, yield a poor correlation (R^2^ = 0.04, n = 70, p = 0.06, the result is not shown). But a scatter plot between these two parameters shows two distinct trends as represented in Fig. 3c. Relatively, a small set of data points (red circles) is characterised by a reasonably high degree of correlation (R^2^ = 0.71, n = 25, p = 0.0001) and a large slope (−6.95) representing strong raindrop evaporation. The other set of data points (black circles) is characterised by a low correlation coefficient (R^2^ = 0.18, n = 40, p = 0.003), and a much lower slope (−0.37) in this case represents considerably reduced raindrop evaporation.

We have also examined the rainfall records and their oxygen isotopic values of the IITM and the Dhankawadi sites. Figure S2a shows a scatter plot of the rainfall variations over these two sites; only those days were considered when it rained more or less synchronously at both the sites. Figure S2b shows the correlation between the isotopic records of the corresponding rain events. Rainfall data at these two sites exhibited a correlation of R^2^ = 0.57 (n = 51, p < 0.0001). But the corresponding isotopic records reveal a significantly better correlation (R^2^ = 0.73, n = 51, p < 0.0001) implying that the isotopic variability behaved more coherently than the rainfall. This coherent behaviour arises from the fact that the rainfall in these two places originated from the same cloud system. The isotopic composition of rainfall is more controlled by the moisture dynamics rather than the rainfall event^29^, hence they respond more to larger scale processes and remain more resilient than the rainfall which is sensitive to micro-climatic conditions. In other words, it could be understood that the meteorological parameters, the rainfall, even within a spatial scale of ca. 15 km is highly susceptible to environmental variability, giving rise to spatial heterogeneity. However, the isotopic values of rainfall are relatively insensitive to micro-environmental effects. This means that a reasonable amount of raindrop evaporation at the IITM site (in the year 2016) does not result in a significant amount of isotopic variability relative to the Dhankawadi site which experienced a much smaller amount of raindrop evaporation.

### Isotopic characteristics of transpired water

Here we discuss the isotopic characteristics of rainfall vis-à-vis the transpired water as shown in Fig. 1. It is believed that leaves operate under isotopic steady state so the biotically mediated vapour loss is generally non-fractionating, a proposition initially proposed in 1970^30^. Subsequently, it was demonstrated by the other investigators^31^. The soil water is sourced from rainwater, fog and groundwater. However, fog formation during the monsoon season is negligibly small and groundwater level stays at relatively higher depth (>60 cm). In addition, the groundwater has higher residence time compared to the processes that occur on an intra-seasonal time scale. Hence, in this case, the rainwater is the only major source for the soil water which, in turn, suffers from isotopic fractionation due to evaporation and hence likely to be isotopically enriched relative to the rain water^31^. This soil water absorbed by the plant and transpired through the leaves is also expected to retain that enriched state and maintain a positive offset (i.e., δ^18^O_Tr_ − δ^18^O_Rn_ > 0)^21^. Our observations, generally reveal that δ^18^O_Tr_ is indeed higher than δ^18^O_Rn_ (see Fig. 1), but this offset is observed to have considerable variability. We suggest that δ^18^O_Tr_ is mainly governed by the soil evaporation processes, which in turn, are controlled by the changes in environmental conditions such as relative humidity, temperature, and wind speed.

Examination of Fig. 1 shows that the difference in δ^18^O_Tr_ and δ^18^O_Rn_ is very significant during the low rainfall regime and less pronounced during the high rainfall regime. This could be explained in terms of soil water evaporation. Soil water, including the leaf water, oxygen isotopic compositions are determined primarily by the δ^18^O of precipitation^32^ followed by subsequent isotopic fractionation during evaporation and diffusion^5,24^. Hence, the strengthened evaporation process during the reduced humidity conditions, would produce isotopically depleted water vapour into the atmosphere, but make the remaining soil water heavier in isotopic composition which is ingested by trees and plants. Hence, the plant-transpired water is usually isotopically enriched than the soil-evaporated water; this characteristic feature has been used to partition the evaporated vapour from the transpired vapour^33^.

In our case, the soil water experiences high evaporative enrichment during the low rainfall phases, but much less enrichment during the high rainfall regime. Since the low rainfall phase is characterised by reduced humidity, evaporation of soil water and in turn, the subsequent isotopic enrichment of the transpired water would be predominant during the low rainfall regime. This is expected to result in an inverse correlation between the relative humidity (RH) and δ^18^O_Tr_. On the other hand, a warmer environment (i.e. higher T) would accelerate soil water evaporation, thereby enhancing the isotopic values of the transpired water. Intense wind (W) also facilitates increased evaporation, but its effect is expected to be less on δ^18^O_Tr_ than temperature and humidity. In order to examine the effect of these parameters we have estimated linear correlation coefficients between δ^18^O_Tr_ and RH, T (Table 1a) and W. Average values (RH, T, W) were estimated during which the transpired water was collected and then the correlation coefficients calculated. As expected, the high negative correlation was observed between RH and δ^18^O_Tr_, and the positive correlation was observed between temperature and δ^18^O_Tr_. The correlation between wind speed and δ^18^O_Tr_ was relatively less significant (not shown) implying that the isotopic composition of transpired water is less sensitive to wind speed. This behaviour was examined separately for all the four plants and a consistent result was found in all the cases, the result is summarised in Table 1. In order to examine the effect of soil evaporation process *vis-a-vis* other processes, such as the biomechanical parameters on the isotopic composition of the transpired water, we have calculated linear correlation coefficients between δ^18^O_Tr_ and temperature and relative humidity integrated for 6 hr (sample collection time), 12 hr (only daytime; 6 am to 6 pm) and 24 hr (day and night) time frame. It was observed that the correlation coefficients (see Table 1a) are practically the same for all the three time durations. This exercise clearly demonstrates that the soil evaporation process is the major factor that determines the isotopic composition of transpired water and other mechanisms such as the tree physiological processes or the isotopic exchange between the chamber vapour and the leaf water, specific to our experimental procedure, had negligible effect. The correlation coefficients are also calculated in regard to d-excess values and shown in this Table (1b) will be discussed later.

**Table 1:** Liner correlation coefficients have been calculated between the meteorological parameters (temperature and relative humidity) and the oxygen isotope (Table a) and d- excess values (Table b) of the transpired water for the four potted plants.

**(a)**		δ^18^O P1	δ^18^O P2	δ^18^O P3	δ^18^O P4	**(b)**		d_Tr_ P1	d_Tr_ P2	d_Tr_ P3	d_Tr_ P4
Temperature	6 hr	0.45	0.45	0.45	0.57	Temperature	6 hr	−0.58	−0.56	−0.61	−0.70
	12 hr	0.47	0.46	0.50	0.59		12 hr	−0.59	−0.56	−0.66	−0.71
	24 hr	0.44	0.43	0.48	0.59		24 hr	−0.57	−0.53	−0.66	−0.68
Relative Humidity	6 hr	−0.72	−0.76	−0.66	−0.83	Relative Humidity	6 hr	0.75	0.81	0.62	0.82
	12 hr	−0.73	−0.76	−0.67	−0.83		12 hr	0.76	0.79	0.64	0.83
	24 hr	−0.75	−0.78	−0.71	−0.86		24 hr	0.76	0.79	0.66	0.83

All values are significant to 0.01 levels.

**Figure 4:**
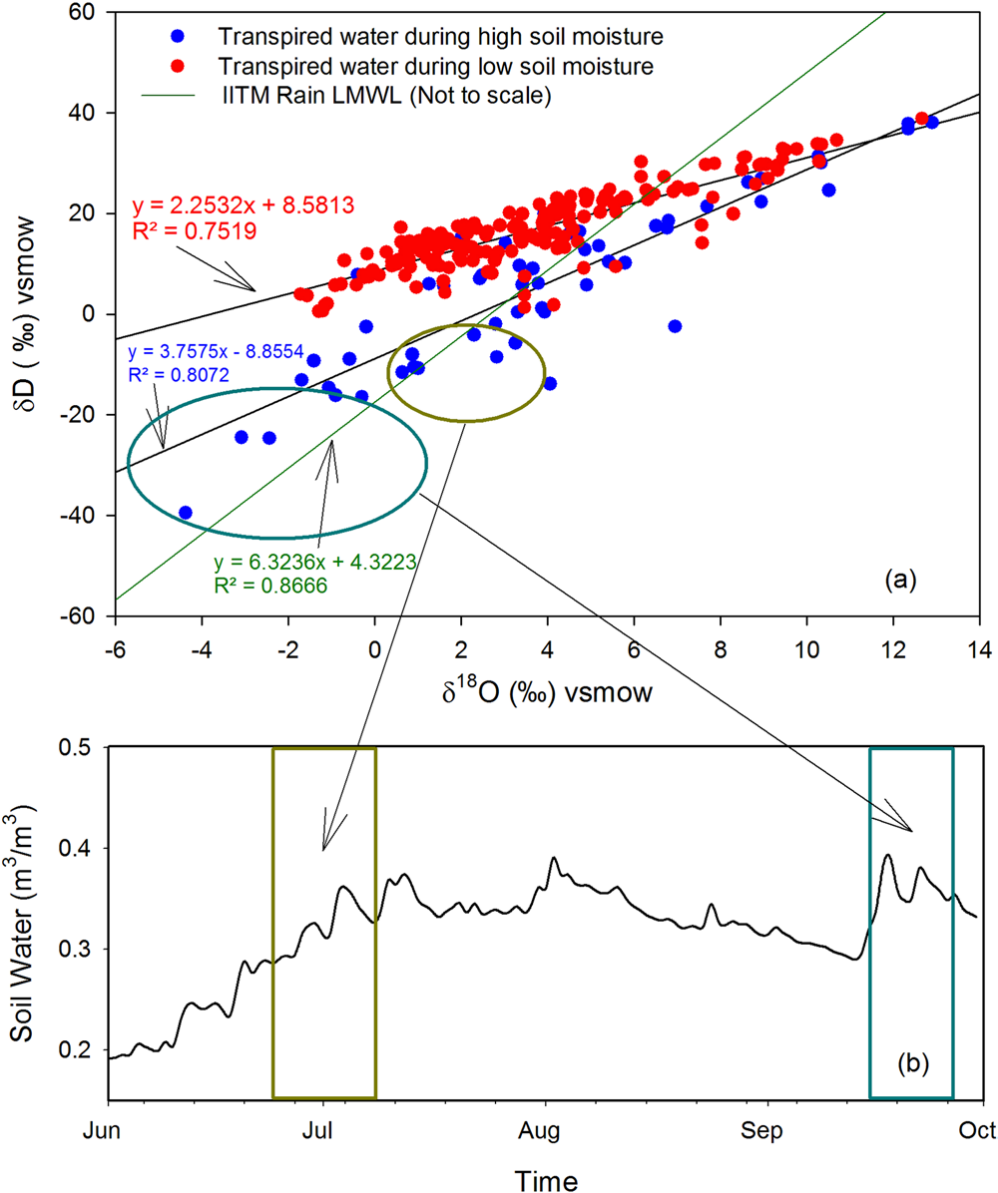
δ^18^O- δD plot for the transpired water during high and low rainfall regime. (**a**) A composite δ^18^O- δD plot of the transpired water, taking into account all the four plants, shown as coloured points. Two distinct trends are apparent as shown by two sets of data points in red and blue respectively. The LMWL of the IITM rainwater (green line; not to scale) is shown for reference. A relatively high slope (3.75) for the transpired water indicates greater soil water content and hence less evaporation; a lower slope (2.25) indicates decreased amount of soil water as a result of increased evaporation. (**b**) A time variation of soil water content based on GLEAM v3 data averaged over a square grid of 1° × 1° around the sampling site. Sharp rise in soil water content (indicated by coloured rectangles) corresponded to low isotopic values in transpired water (circled data points) indicating reduced evaporation. The plot was made using a licensed copy of SigmaPlot.

Further evidence of this assertion comes from the following observations. As mentioned earlier, some points in Fig. 2b (encircled) show a little different behaviour. To investigate this behaviour we have re-plotted this diagram taking into account δ^18^O_Tr_ and δD_Tr_ of all the four plants. Fig.  4a shows such a scatter plot showing all the oxygen and hydrogen isotopic values (in blue and red). One set of data (red points) contains relatively higher isotopic values and yields a regression line with a slope of 2.25. The other set (blue points) is characterised by relatively low isotopic values and its regression line yields a slope of 3.75. This is consistent with the general perception that the evaporated samples are characterised by higher δ^18^O but lower slope when their isotopic values are plotted on a δ^18^O -δD space^31^. This means that the samples represented by red points were subjected to enhanced evaporation (and hence yield a lower slope of 2.25) than the samples denoted by blue points (slope = 3.75). Inverse correlation between transpired water δ^18^O_Tr_ and d-excess (defined as d_Tr_ = δD_Rn_ − 8* δ^18^O_Rn_) also indicates evaporation and a strong correlation (R^2^ = 0.95, n = 188; p < 0.0001, see Supplementary Fig. S3a) for the first set of data indeed supports enhanced evaporation of soil water, which was eventually transpired by the plants.

The second set of data also shows a negative (Fig. S3b) trend, however, the dataset is more scattered, resulting in a slightly reduced correlation (R^2^ = 0.84, n = 56, p < 0.001) indicating the evaporation process is relatively less. A large proportion of red points in Fig. 4a (relative to blue points) also indicate that the low rainfall regime is more predominant than the high rainfall regime during the entire monsoon season.

We have also tested the above-mentioned hypothesis in the case of the natural plant studied in the year 2017. Firstly, the δ^18^O_Tr_ − δD_Tr_ slope (5.72) for the natural plant is much higher than the potted plant (3.02), implying that the natural soil is subjected to much less evaporation than the pot soil because of its large dimension (Fig. 2b, d). Secondly, a scatter diagram between the transpired water δ^18^O_Tr_ and the corresponding d-excess (d_Tr_) values in the natural plant also depicts a strong inverse correlation as shown in Fig. S4. But a few data points (points marked in blue and encircled in Fig. S4) stay off the general trend and analysis of these events reveal that most of these points belonged to heavy rainfall events in the neighbouring area resulting in subdued soil evaporation during those days. So, barring these heavy rainfall days, the remaining set of data points yield a regression line with a correlation coefficient of R^2^ = 0.73 (n = 100, p < 0.0001) implying the evaporation process, by and large, controlled the isotopic composition of the transpired water. But the correlation coefficient (R^2^ = 0.65) in case of the natural plant is slightly smaller than that observed in the potted plants. This is expected because the pots are placed in a canopy providing certain temperature stability and their small dimensions (soil volume) are subjected to a relatively steady evaporation process. On the other hand, the natural plant being in the open place would experience higher temperature variability and due to the inherent heterogeneity of the soil it is likely to be subjected to a non-linear evaporation process. Nevertheless, a reasonably high correlation coefficient in both the cases, indicates that ≥65% variability in transpired water isotopic composition could be explained by means of soil evaporation process.

Going back to the previous discussion, we see the slopes of these two data sets, [i.e., data points consisting of the red (slope: 2.25) and blue (slope: 3.75) datapoints of Fig. 4a] are also significantly less than the slope of IITM-LMWL (green line; drawn not to scale; slope = 6.32). This implies that the slope of the δ^18^O_Tr_ vs. δD_Tr_ line can have maximum (during intense precipitation) value upto the extent of the LMWL slope, and with increased soil evaporation (during weak precipitation) the slope would be progressively reduced. The extent of soil water evaporation primarily depends on the relative humidity, mediated by rainfall, and additionally on the air temperature, wind and available energy. Higher rainfall would produce increased amount of soil water and hence would reduce evaporation, which in turn, would reduce isotopic fractionation. This water when transpired by plants will also have lower isotopic values and relatively higher slope on a δ^18^O_Tr_ − δD_Tr_ diagram. An opposite behaviour was observed for low rainfall scenario. To have a further check, we have examined the top surface soil water content (0–10 cm) using the GLEAM v3 data set^34,35^. Figure 4b shows the time profile of soil water content averaged over an area of 0.5° lat × 0.5° lon around the sampling site. A sharp increase in soil water content in mid to late September indicated by a blue rectangular box, is well-manifested in the lowest isotopic values of the transpired water (encompassing by a bigger ellipse in Fig. 4a). Similarly, another increasing trend in soil water content around July (indicated by yellow rectangular box in Fig. 4b) is also captured by the transpired water isotopic values falling approximately in the middle of the lower regression line, as labeled by a smaller ellipse. This observation may be summarised as follows: higher soil water would result in higher slope on a δ^18^O_Tr_ − δD_Tr_ plot of plant transpired water; and similarly, lower soil water would result in a smaller slope on a δ^18^O_Tr_ − δD_Tr_ plot of plant transpired water. Hence the slope of the transpired water line may be considered as a qualitative measure of the extent of soil water loss through evaporation. A probable application of this proposition could be to carry out real-time isotopic analysis of the transpired water, such as by laser spectroscopy and determine the evaporation line on the daily scale. The variability of the slope of these lines could be used to quantify the extent of soil water evaporation on intraseasonal time scales. This may provide an alternative means to study the soil water evaporation process using the isotopic technique.

### Intraseasonal variation of deuterium excess at different phases of monsoon

**Figure 5:**
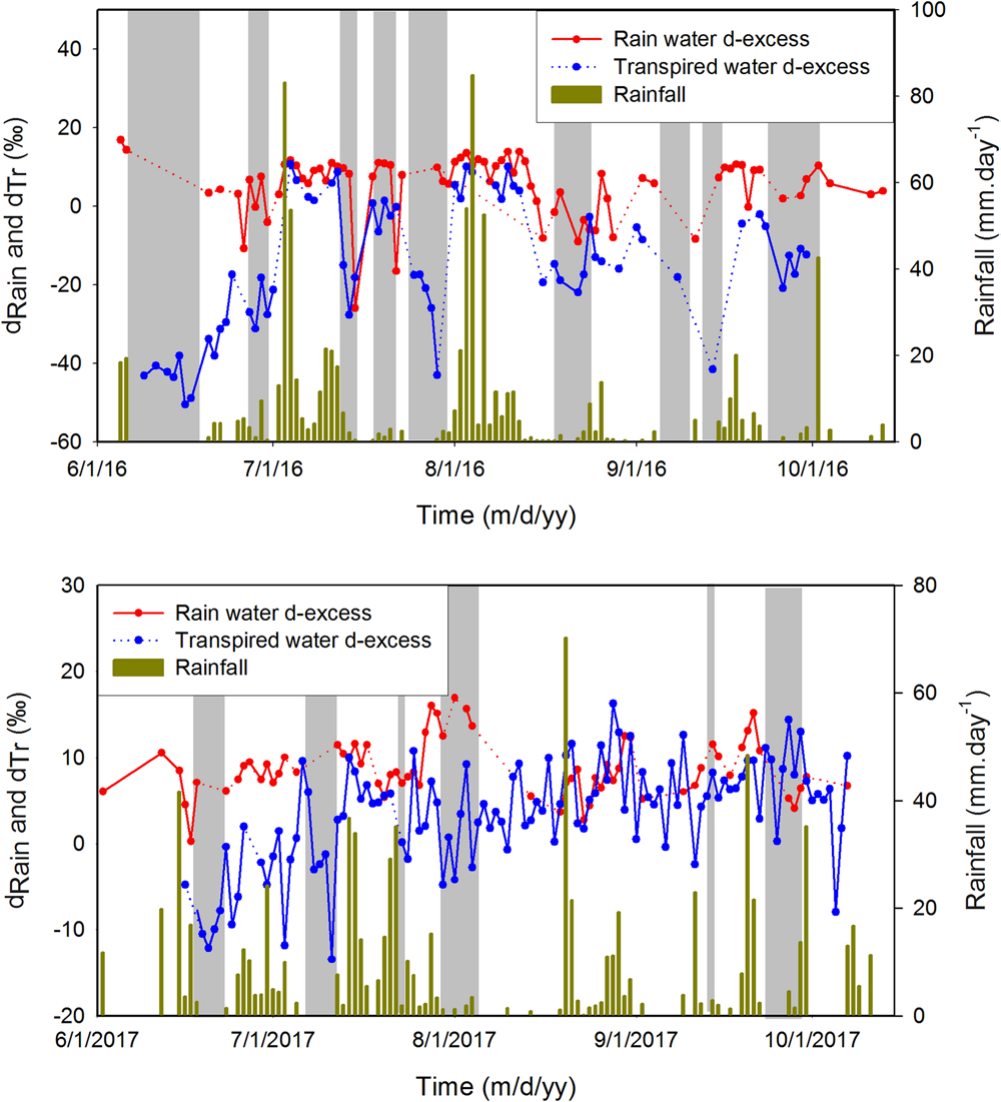
The d-excess time series. Time profile of rainwater d-excess (red line) and transpired water d-excess (blue line) in 2016. The bar diagram represents the rainfall variability. The vertical shadings show the periods of low rainfall during which the difference between the d-excess of rain water and transpired water is generally high compared to the rainy days when this difference is generally small. The lower panel diagram shows the same features for 2017. The plot was made using a licensed copy of SigmaPlot.

The deuterium excess is known to have a higher dependency on the relative humidity and as a result, strongly responds to evaporation process. Since the transpired water as demonstrated in this work mimics the soil water evaporation process, we examine the d-excess variability of the rain (d_Rn_) in association with the transpired water. Fig. 5 shows the d-excess of rainwater (blue line), transpired water (red line) and additionally, the rainfall data (dark yellow bar). Results for both the potted plant (top panel) and natural plant (bottom panel) are shown. Deuterium excess is mainly controlled by temperature and relative humidity of the air mass over the moisture source surface, although the wind speed at the source also plays a role^36^. When surface water evaporates its deuterium content is reduced compared to heavier oxygen^28^ making the d-excess value of surface water depleted. As explained earlier, the transpired water inherits the characteristics of the evaporated soil water, so its d-excess is expected to be less than that of the rain water. This is indeed observed in this diagram; d_Tr_, in general, is systematically lower than d_Rn_. This characteristic feature is prominent during the low rainfall regime as shown in the Fig. 5 (vertical shading). But when the rainfall is relatively high, the humidity is increased, so the soil evaporation and in turn the transpiration process weakens. Hence, during the heavy rainfall regime, the difference between d_Rn_ and d_Tr_ is reduced, as illustraded by the non-shaded regions in Fig. 5a,b.

Similar to δ^18^O_Tr_, we have also examined the dependency of d_Tr_ with the environmental parameters, such as RH and T (Table 1b). Strong positive correlation (R^2^ = 0.64, n = 64, p = 0.0001) was found in case of RH vs. d_Tr_ and negative correlation (R^2^ = 0.47, n = 64, p = 0.0001) between temperature and d_Tr_ as summarised in Table 1b. Since lower humidity promotes evaporation, d-excess of surface water is reduced^28^ making it positively correlated with humidity; but opposite behaviour is noticed with temperature. The implication of this variation is that deuterium excess of rain and transpired water at a given place could provide an alternative means to delineate the humid, and especially, the dry phases of monsoon on the intra-seasonal time scales.

**Figure 6:**
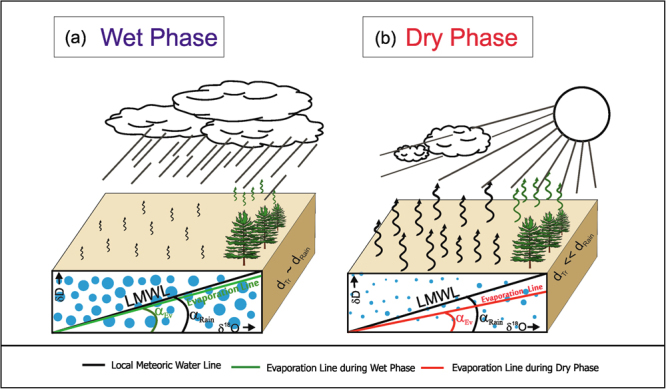
A conceptual model of the effect of soil water evaporation on transpiration isotope: A conceptual representation of the evaporation line on δ^18^O-δD isotope diagram and its relationship with that of the meteoric water line during (**a**) humid and (**b**) dry phases of monsoon. (**a**) Humid phase is characterised by heavy rainfall, low soil evaporation as well as transpiration (small wavy arrows), and relatively high soil water (large blue circles). The soil surface cross section shows the δ^18^O - δD space on which the meteoric water line (slope = α_Rain,_ in black) has been drawn.The evaporation line (green line) is also shown whose slope (α_Ev_) is slightly lower than that of the meteoric water line. The difference between d-excess of the evaporation line and that of the meteoric water line is also small. (**b**) Dry phase is represented by sunny days, low rainfall, high evaporation and transpiration (heavy wavy arrows), low soil water (tiny blue circles). The slope of the meteoric water line (α_Rain_) is relatively much higher than the corresponding evaporation line (α_Ev,_ in red) as compared to the humid phase. The d-excess of the transpired water is much smaller than that of the meteoric water. The graphics were made using licensed CorelDRAW.

A conceptual model of these processes has been schematically shown in Fig. 6. We consider two extreme scenarios: a high rainfall situation (Wet Phase) resulting in a near-saturation of ambient water vapour, moderate temperature and calm wind condition. In this case the soil will have high water content but weak evaporation and plant transpiration, as shown in Fig. 6a (small wavy arrows). The other case is characterised by little rainfall, increased sunshine, low humidity, relatively higher temperature and higher wind speed (Dry Phase). This situation will intensify evaporation and transpiration as indicated by heavy wavy arrows (Fig. 6b). In the first case, the oxygen isotopic values of transpired water would approach the value of the local rainwater; the slope and the d-excess (d_Tr_) value of the evaporation line on a δ^18^O − δD diagram would also approach the corresponding values of the rain water. In the second case, increased evaporation would result in isotopically enriched soil water, and in turn enriched transpired water. This will result in a lower slope of the δ^18^O_Tr_ − δD_Tr_ evaporation line, hence it will deviate from that of the meteoric water line as shown in Fig. 6b. In regard to the d-excess, the first case would yield similar d-excess values for rainwater and transpired water, while in the second case, the d-excess value of the transpired water would be less than that of the rainwater, as shown in Fig. 6. These kinds of environmental conditions are typically experienced during the active and break phases of monsoon, an important attribute of the Indian monsoon system^37^. Study of the sub-seasonal rainfall variability characterising the active and break periods is an active field of monsoon research because of its societal and economic impact^38^. Therefore, prediction of intraseasonal variations and of the occurrence of break periods, and in particular, their duration and intensity, is very important^39^. This requires identification of break periods based on the characteristic variations of meteorological variables, such as rainfall (see ref.^37^ for a review), wind strength^40^, convection and 850 hPa zonal winds^41^, or outgoing long wave radiation (OLR) anomalies^42^. However, almost all of these methods rely on atmospheric variables, but do not take into account any biophysical parameter, such as plant transpiration. Secondly, these methods calculate large-scale meteorological fields, thus define the break and active periods over a very large area. Local or regional scale features may not be adequately represented, yet such information is important because prolonging break conditions may adversely affect the agricultural output^38^. The isotopic analysis of transpired water as demonstrated in this work may fill this gap. Since plant transpiration and its isotopic composition strongly respond to moisture stress conditions, both in the soil as well as in the ambient environment, the isotopic method seems to be more appropriate for detecting dry phases of the monsoon. In this context, it may be mentioned that the soil moisture measurements also provide valuable information about the hydrological system, but the isotopic analysis as demonstrated in this work (also see the Method section) could complement the soil moisture analysis for a better understanding of the hydrological processes.

## Conclusions

Isotopic analysis of plant transpired water was carried out during the summer monsoon seasons of 2016 and 2017. The isotopic variability of plant transpired water was observed to be mainly controlled by the soil water dynamics. Evaporation of soil water resulted in the isotopic enrichment and this enriched state is maintained in the water transpired by the trees and plants. The evaporation of soil water primarily depended on humidity which in turn was modulated by rainfall. Low rainfall regime reduced the ambient humidity which intensified soil water evaporation resulting in isotopically enriched soil water, and in turn, in the isotopically enriched transpired water. During the dry periods, oxygen isotopic compositions of the transpired water showed relatively higher values while its d-excess values became negative relative to the rainwater. During the wet phases of monsoon these differences steadily diminished and the oxygen isotope and d-excess values of the transpired water approached the corresponding rainwater values. Since such behaviour can be observed on a small spatial scale, this characteristic feature of plant transpired water could be implemented to identify the break phases of monsoon on both local and regional scales.

## Data and Methods

Potted plants with large leaf size were chosen for this experiment. Plants planted in four pots were: (i) *Ficus benjamina* L, (ii) *Schefflera actinophylla*, (iii) *Codiaeum variegatum*, and (iv) *Ficus rubiginosa*. The plants have been labeled as P1, P2, P3 and P4 respectively. Each plant had several branches, with total number of leaves varying from 150 to 400. A few branches of leaves of each of them were selected and intercepted water, if any, were removed, then these were placed into a transparent plastic bag and locked with cable ties to create an approximately isolated system, termed as a *chamber*. The system was left for about 6 hours in order to accumulate enough amount of transpired water. Afterwards, the transpired water was collected and transferred to a plastic vial for isotopic analysis. The average amount of transpired water collected was 2.3 ± 1.4 ml from each plant per day. The amount of co-existing vapour was estimated considering 100% saturation and found to be negligibly small (by weight) relative to the liquid water (result not shown). A total of 271 transpired water samples was collected during the study period from 7^th^ June to 30^th^ September during the summer monsoon season of 2016. The same experiment was continued during the monsoon season of 2017 on a natural plant (*Cassia fistula L*) which was slightly bigger in size than the potted plants. The sampling period was 16^th^ June to 5^th^ Oct and a near-continuous (107 samples out of 112 days) time series of δ^18^O_Tr_ was generated. The average amount of transpired water collected was 10.3 ± 4.5 ml per day. The collection technique was similar to that of Menchaca *et al*.^21^ except for the duration of the collection. Menchaca *et al*.^21^ had collected for an integrated period of 24 hours, but we chose to restrict this to about 6 hours during the daytime for the following reasons. The transpiration process usually picks up in the morning hours; the rapid turnover of water in transpiring leaves means that the signature of transpiration is usually similar to the isotopic composition of plant source water, especially during mid-day^43^. (ii) While some isotopic enrichment can occur in the leaf due to the same kinetic and diffusive effects that lead to evaporative fractionation in soils^44^, these non‐steady‐state leaf‐scale effects usually occur only during early morning hours and late afternoon^15,17,26,43–45^. (iii) Hence, in order to achieve an isotopic steady state condition of the transpired water, the 1000–1600 hour timeframe was chosen for this experiment. In addition to the transpired water, we have also collected rainwater near the experimental site (ca. 20 m off the canopy) to determine the isotopic composition of the rainwater that was expected to be ingested by the plants. Additionally, daily rainwater was also collected (only in 2016) from a site- Dhankawadi in the Pune city, situated about 14 km south of the IITM campus. This was done to study the regional variability or the coherent nature of the rainfall and its isotopic characteristics. Rainwater was collected using ordinary rain gauges integrated for 24 hours at 9:00 am throughout the monsoon season.

The collected water was then transferred to leakproof 30 ml plastic bottle and the rainfall amount was measured using a calibrated cylinder. About 55 rain samples were collected from each site in 2016 and 70 samples at IITM in 2017. The weather data (temperature, RH and wind speed) were collected from an automatic weather station (AWS) located just outside the canopy.

The isotopic analysis was performed using an LGR water isotope analyzer (Model: IWA 45 EP) at the Stable Isotope Laboratory of the IITM, Pune, India. Analytical results were reported in standard δ notation defined as follows:

M1 $${\delta }^{18}{\rm{O}}=[{({}^{18}{\rm{O}}/{}^{16}{\rm{O}})}_{{\rm{S}}{\rm{a}}{\rm{m}}{\rm{p}}{\rm{l}}{\rm{e}}}-{({}^{18}{\rm{O}}/{}^{16}{\rm{O}})}_{{\rm{R}}{\rm{e}}{\rm{f}}{\rm{e}}{\rm{r}}{\rm{e}}{\rm{n}}{\rm{c}}{\rm{e}}}]\ast 1000(\textperthousand ,\,{\rm{V}}{\rm{S}}{\rm{M}}{\rm{O}}{\rm{W}})$$

The Reference material is the Standard Mean Ocean Water whose absolute ^18^O/^16^O ratio is 2005 × 10^−6^ (ref.^46^). The measurement precisions are 0.8‰ and 0.08‰ for δD and δ^18^O respectively. In this context, it may be noted that the determination of the isotopic ratio is done on a molecular scale and hence it has potential to provide high resolution data. It is not possible to obtain a similar kind of resolution in case of rainfall measurement by using a single rain gauge or an automatic weather station; a suite of rain gauge equipment consisting of several devices is required to achieve that kind of observational precision. This concept could also be explained in a different manner. The spectrum of rainfall variability is usually much higher than that of the isotopic variability. We have calculated the mean and standard deviations of the rainfall and the corresponding δ^18^O values of the dataset presented in this paper. But since the isotopic values are reported in relative scale, the rainfall values have also been expressed in a similar manner. To make this conversion, we have calculated the climatological daily rainfall value (6.95 mm.day^−1^) for the last 150 years (1865–2015) for the June-Sep (JJAS) season. The relative rainfall value is calculated as follows:

$${\rm{Relative}}\,{\rm{rainfall}}=[({{\rm{R}}}_{{\rm{i}}}-{{\rm{R}}}_{{\rm{clim}}})/{{\rm{R}}}_{{\rm{clim}}}]\ast 1000.$$

Where R_i_ is the rainfall data on the i^th^ day, and R_clim_ is the climatological value of the daily rainfall during the JJAS season. The factor 1000 has been introduced in order to match the definition of the isotopic ratio (See Eq. M1). With this consideration, the mean and standard deviation of both the parameters are calculated and shown in Table 2. It is clear from these calculations that rainfall variability is more than one order of magnitude higher than the corresponding isotopic variability. Hence the isotopic analysis is likely to offer a better means to capture the inherent variability.

**Table 2:** Statistical characteristics of rainfall and their isotopic variabilities observed for the two sites.

	Site: Dhankawadi		Site: IITM			
	Rainfall-2016	δ^18^O-2016	Rainfall-2016	δ^18^O-2016	Rainfall 2017	Rainfall δ^18^O
Mean	−95.22	−0.68	−121.9	−0.38	587.35	−2.40
Standard deviation	959.41	2.33	892.5	3.08	1878.9	3.89

Here the parameter “rainfall” is defined as relative deviation from a climatological mean daily rainfall value and divided by the same and then multiplied by 1000. With this consideration the mean and standard deviations are calculated for the two sites rainfall and their corresponding oxygen isotopic records. It is evident that the standard deviation of rainfall variability is more than one order of magnitude higher than that of the isotopic variabilities.

And secondly, the water isotopic values, such as of the rainfall, almost always respond better to large scale changes rather than local scale processes as observed by several investigators^47^. Because of these reasons, the isotopic analysis of transpired water is likely to provide better quantitative information about the soil evaporation process than the conventional process of measuring the soil water content using the lysimeter.

## Electronic supplementary material

Supplementary information

Dataset 1
