# Supplementary material for: Isotopic study of intraseasonal variations of plant transpiration: an alternative means to characterise the dry phases of monsoon
Source: Sci Rep. 2018 Jun 5;8:8647. doi: 10.1038/s41598-018-26965-6 (PMC5988688; doi:10.1038/s41598-018-26965-6)
Supplement: Supplementary file 1 — Supplementary information [file 41598_2018_26965_MOESM1_ESM.docx]

Supplementary materials

MS No. SREP-17-39105

**Isotopic study of intraseasonal variations of plant transpiration: an alternative means to characterize the dry phases of monsoon**

S. Chakraborty^1,2^, A. R. Belekar^2^, A. Datye^1^, N. Sinha^1^

^1^Indian Institute of Tropical Meteorology, Pune, India

^2^Savitribai Phule Pune University, Pune, India


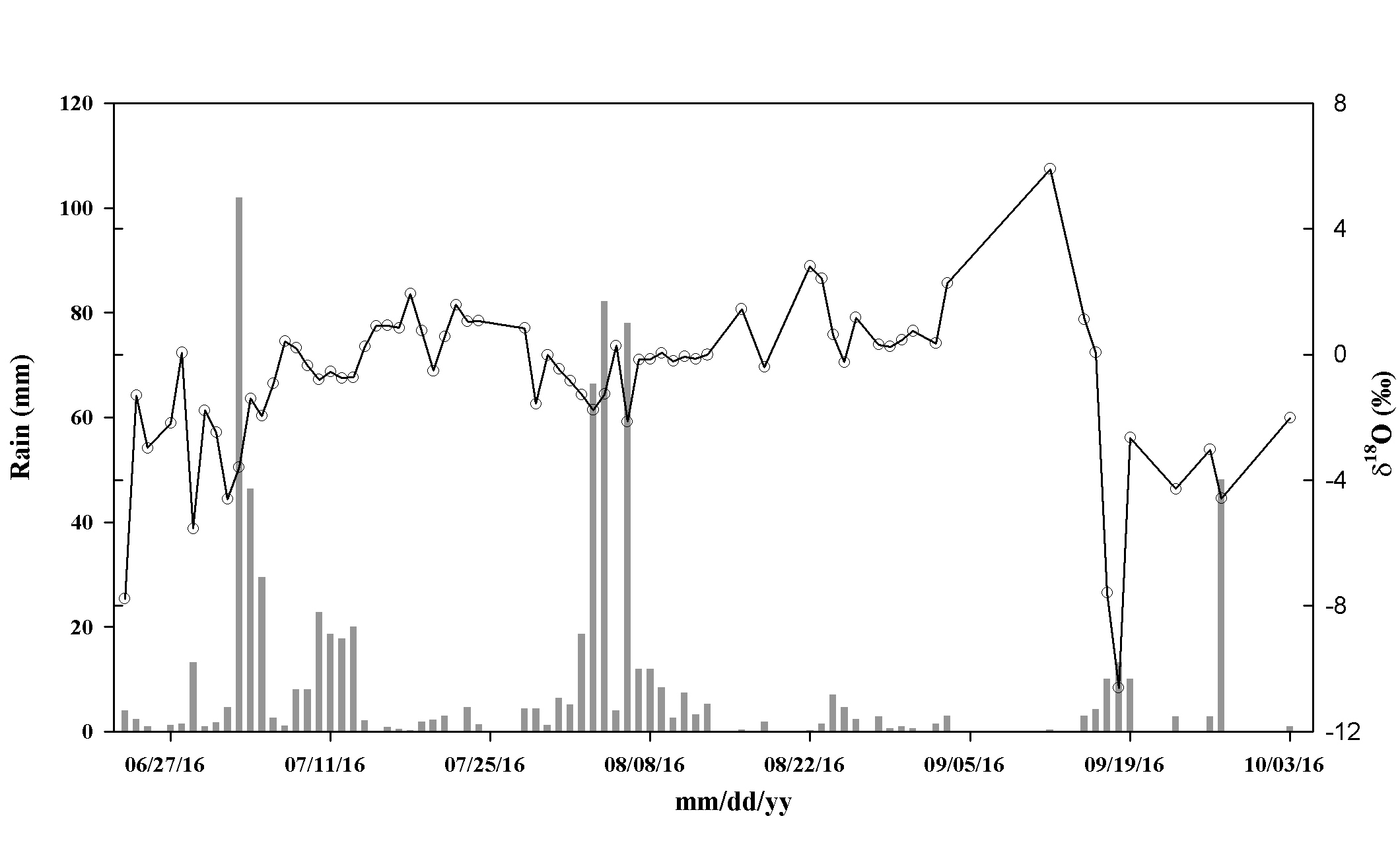


Figure S1: The rainfall (bar) and its oxygen isotopic (line) time series for the Dhankawadi site. The plot was made using licensed copy of SigmaPlot.


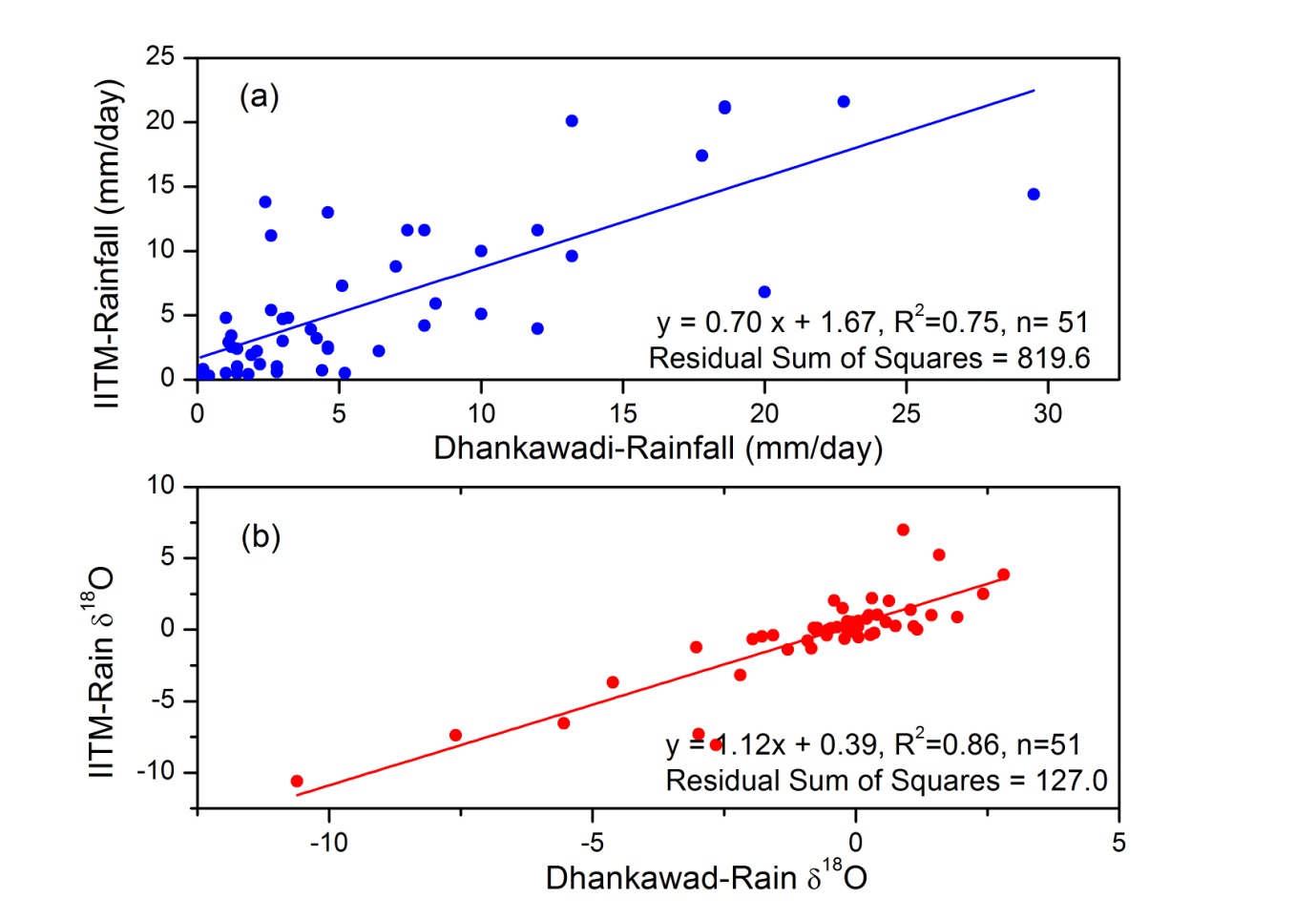


Figure S2: The correlation between (a) rainfall and their (b) isotopic values at the Dhankawadi and IITM site. The rainfall pattern shows more scatter, while their isotopic values seem to be better constrained. The plot was made using licensed copy of Origin 8.5.


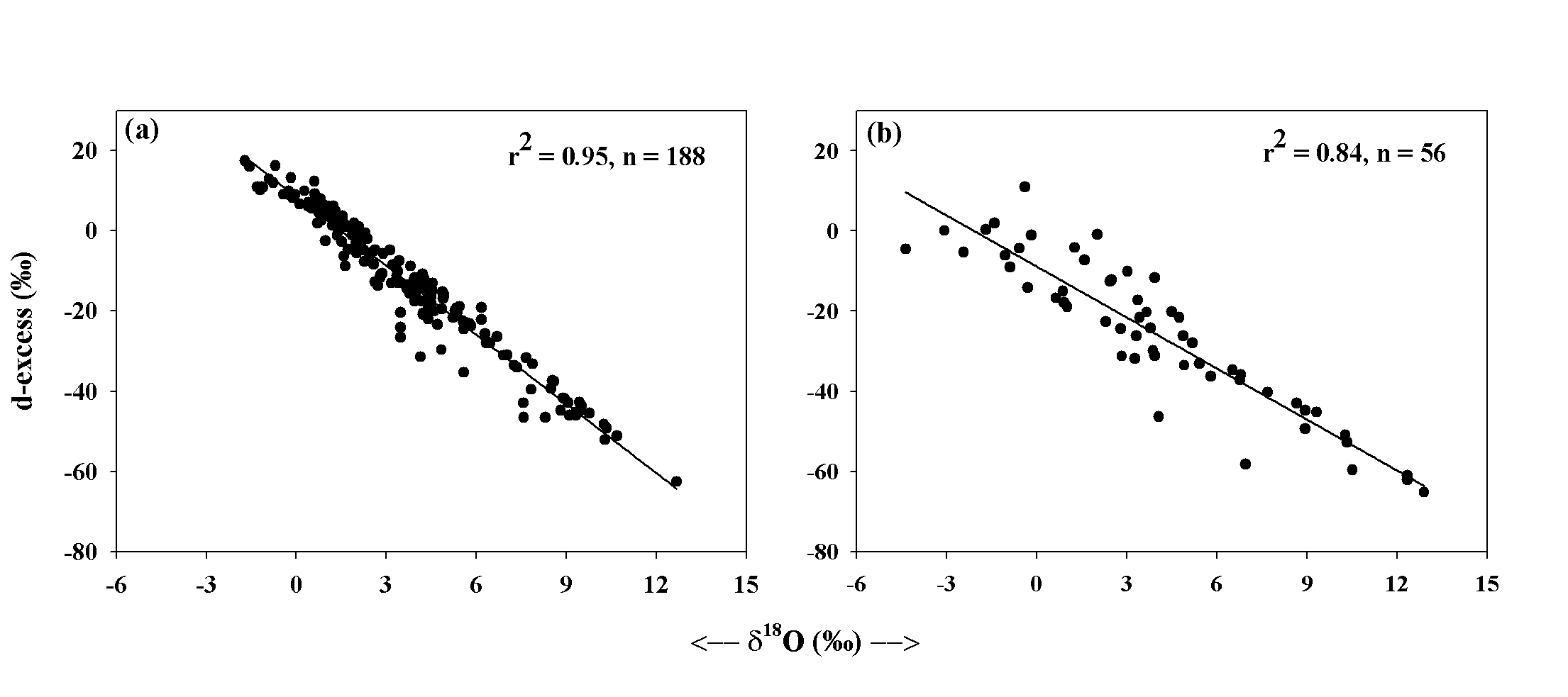


Figure S3. Relationship between the d18O and the d-excess of the transpired water. a) A stronger correlation represents increased soil water evaporation, while b) a relatively weaker correlation indicates reduced evaporation effect. Plot made using licensed copy of SigmaPlot.


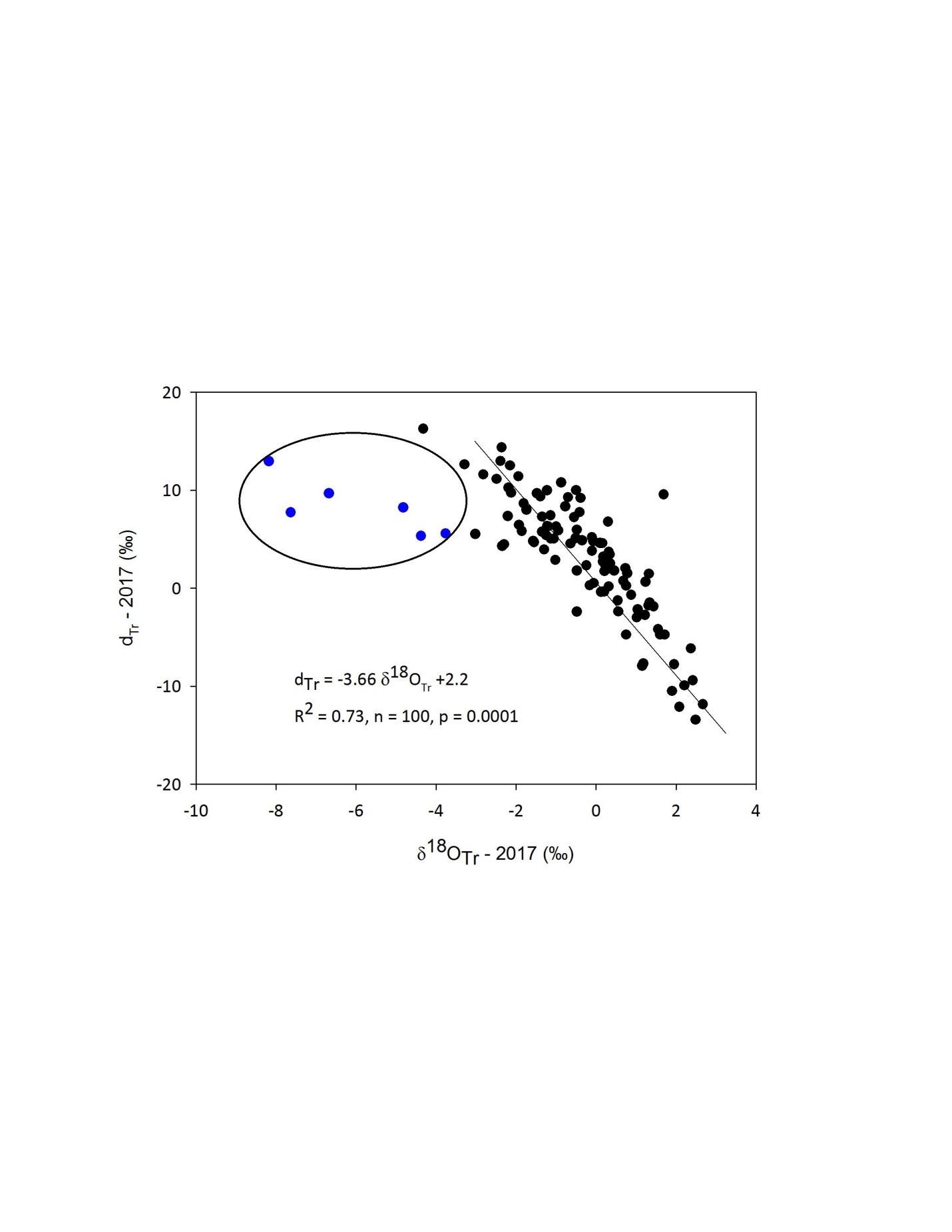


Figure S4. The relationship between d-excess and d18O of transpired water for the natural plant. Though a strong inverse correlation is found similar to the potted plant as shown above some points (encircled) deviate from the main trend. These point belong to relatively heavy rainfall events.
