# Supplementary material for: Isotopic study of intraseasonal variations of plant transpiration: an alternative means to characterise the dry phases of monsoon
Source: Sci Rep. 2018 Jun 5;8:8647. doi: 10.1038/s41598-018-26965-6 (PMC5988688; doi:10.1038/s41598-018-26965-6)
Supplement: Supplementary file 2 — Dataset 1 [file 41598_2018_26965_MOESM2_ESM.docx]

**Supplementary Dataset:**

**Rainfall and its isotopic values for the two sites- Dhankawadi and the IITM Campus**

**Isotopic study of intraseasonal variations of plant transpiration: an alternative means to characterize the dry phases of monsoon**

S. Chakraborty^1,2^, A. R. Belekar^3^, A. Datye^1^, N. Sinha^1^

^1^Indian Institute of Tropical Meteorology, Pune, India

^2^Department of Atmospheric and Space Science, Savitribai Phule Pune University, Pune, India

^3^Department of Environmental Science, Savitribai Phule Pune University, Pune, India

|  | SITE: Dhankawadi | | SITE: IITM | |  |
| --- | --- | --- | --- | --- | --- |
| Date | Rainfall (mm/day) | δ^18^O (‰) vsmow | Rainfall (mm/day) | δ^18^O (‰) vsmow |  |
| 25-06-2016 | 1 | -2.98 | 4.8 | -7.32 |  |
| 27-06-2016 | 1.2 | -2.19 | 3.4 | -3.16 |  |
| 28-06-2016 | 1.4 | 0.05 | 1 | 0.60 |  |
| 29-06-2016 | 13.2 | -5.54 | 9.6 | -6.56 |  |
| 30-06-2016 | 1 | -1.78 | 0.5 | -0.46 |  |
| 02-07-2016 | 4.6 | -4.61 | 13 | -3.69 |  |
| 05-07-2016 | 29.5 | -1.96 | 14.4 | -0.66 |  |
| 06-07-2016 | 2.6 | -0.92 | 5.4 | -0.78 |  |
| 07-07-2016 | 1.1 | 0.41 | 2.9 | 1.05 |  |
| 08-07-2016 | 8 | 0.21 | 4.2 | 0.77 |  |
| 09-07-2016 | 8 | -0.35 | 11.6 | 0.17 |  |
| 10-07-2016 | 22.8 | -0.80 | 21.6 | 0.12 |  |
| 11-07-2016 | 18.6 | -0.55 | 21.1 | -0.38 |  |
| 12-07-2016 | 17.8 | -0.75 | 17.4 | -0.04 |  |
| 13-07-2016 | 20 | -0.73 | 6.8 | 0.11 |  |
| 14-07-2016 | 2.1 | 0.25 | 2.2 | 1.02 |  |
| 15-07-2016 | 0.1 | 0.90 | 0.5 | 6.99 |  |
| 18-07-2016 | 0.2 | 1.93 | 0.5 | 0.88 |  |
| 19-07-2016 | 1.9 | 0.75 | 1.9 | 0.27 |  |
| 20-07-2016 | 2.2 | -0.52 | 1.2 | -0.02 |  |
| 21-07-2016 | 3 | 0.57 | 3 | 0.52 |  |
| 22-07-2016 | 0.1 | 1.58 | 0.2 | 5.24 |  |
| 23-07-2016 | 4.6 | 1.04 | 2.55 | 1.39 |  |
| 29-07-2016 | 4.4 | -1.57 | 0.7 | -0.38 |  |
| 30-07-2016 | 1.2 | -0.02 | 2.55 | -0.13 |  |
| 31-07-2016 | 6.4 | -0.47 | 2.2 | 0.10 |  |
| 01-08-2016 | 5.1 | -0.84 | 7.3 | -1.31 |  |
| 02-08-2016 | 18.6 | -1.29 | 21.2 | -1.38 |  |
| 05-08-2016 | 4 | 0.28 | 3.9 | -0.36 |  |
| 07-08-2016 | 12 | -0.16 | 3.95 | 0.57 |  |
| 08-08-2016 | 12 | -0.16 | 11.6 | 0.52 |  |
| 09-08-2016 | 8.4 | 0.05 | 5.9 | 0.19 |  |
| 10-08-2016 | 2.6 | -0.22 | 11.2 | -0.63 |  |
| 11-08-2016 | 7.4 | -0.06 | 11.6 | 0.53 |  |
| 12-08-2016 | 3.2 | -0.15 | 4.8 | -0.17 |  |
| 13-08-2016 | 5.2 | -0.01 | 0.5 | -0.21 |  |
| 16-08-2016 | 0.4 | 1.44 | 0.3 | 1.02 |  |
| 18-08-2016 | 1.8 | -0.41 | 0.4 | 2.03 |  |
| 22-08-2016 | 0.2 | 2.81 | 0.8 | 3.85 |  |
| 23-08-2016 | 1.4 | 2.42 | 2.4 | 2.51 |  |
| 24-08-2016 | 7 | 0.63 | 8.8 | 2.02 |  |
| 25-08-2016 | 4.6 | -0.25 | 2.4 | 1.51 |  |
| 26-08-2016 | 2.4 | 1.16 | 13.8 | 0.00 |  |
| 28-08-2016 | 2.8 | 0.31 | 0.6 | 2.21 |  |
| 02-09-2016 | 1.4 | 0.35 | 0.5 | -0.24 |  |
| 15-09-2016 | 3 | 1.10 | 4.7 | 0.24 |  |
| 16-09-2016 | 4.2 | 0.05 | 3.2 | -0.53 |  |
| 17-09-2016 | 10 | -7.59 | 10 | -7.40 |  |
| 18-09-2016 | 13.2 | -10.61 | 20.1 | -10.59 |  |
| 19-09-2016 | 10 | -2.65 | 5.1 | -8.07 |  |
| 26-09-2016 | 2.8 | -3.03 | 1.00 | -1.24 |  |
|  | | | | |  |
| SITE: IITM | | | | | |
| Date | Rainfall (mm/day) | δ^18^O (‰) vsmow | Date | Rainfall (mm/day) | δ^18^O (‰) vsmow |
| 02-06-2017 | 11.76 | 0.71 | 25-08-2017 | 1.90 | -0.01 |
| 12-06-2017 | 19.80 | -3.82 | 26-08-2017 | 2.50 | -2.31 |
| 15-06-2017 | 41.60 | -3.15 | 27-08-2017 | 11.00 | -1.19 |
| 16-06-2017 | 3.60 | -0.11 | 28-08-2017 | 11.20 | -1.62 |
| 17-06-2017 | 16.90 | 0.18 | 29-08-2017 | 19.30 | -9.25 |
| 18-06-2017 | 2.60 | 0.79 | 30-08-2017 | 3.70 | -9.06 |
| 23-06-2017 | 1.50 | 1.02 | 31-08-2017 | 6.80 | -2.00 |
| 25-06-2017 | 7.65 | 0.24 | 02-09-2017 | 2.20 | 0.97 |
| 26-06-2017 | 12.40 | -0.50 | 09-09-2017 | 3.90 | -4.93 |
| 27-06-2017 | 10.30 | -0.29 | 11-09-2017 | 23.00 | -5.22 |
| 28-06-2017 | 3.90 | 0.61 | 12-09-2017 | 2.30 | -5.43 |
| 29-06-2017 | 4.00 | 0.01 | 14-09-2017 | 2.90 | -9.33 |
| 30-06-2017 | 24.25 | -0.87 | 15-09-2017 | 2.00 | -8.15 |
| 01-07-2017 | 4.95 | 0.67 | 17-09-2017 | 1.40 | -2.20 |
| 02-07-2017 | 4.50 | 0.48 | 19-09-2017 | 7.90 | -10.70 |
| 03-07-2017 | 10.00 | -0.22 | 20-09-2017 | 48.50 | -9.84 |
| 05-07-2017 | 2.40 | 0.78 | 21-09-2017 | 21.60 | -4.74 |
| 12-07-2017 | 7.65 | -2.42 | 22-09-2017 | 2.40 | -3.67 |
| 13-07-2017 | 2.00 | 0.32 | 27-09-2017 | 4.60 | 1.40 |
| 14-07-2017 | 36.80 | -1.17 | 28-09-2017 | 1.60 | 0.94 |
| 15-07-2017 | 34.00 | -0.61 | 29-09-2017 | 13.70 | -0.84 |
| 16-07-2017 | 14.20 | 0.24 | 30-09-2017 | 35.20 | -0.88 |
| 17-07-2017 | 5.50 | 0.04 | 07-10-2017 | 13.00 | -8.56 |
| 19-07-2017 | 6.60 | -3.01 | 08-10-2017 | 16.70 | -11.80 |
| 20-07-2017 | 14.70 | -5.71 | 09-10-2017 | 5.50 | -15.90 |
| 21-07-2017 | 29.20 | -3.20 | 11-10-2017 | 11.25 | -12.11 |
| 22-07-2017 | 35.20 | -3.63 |  |  |  |
| 23-07-2017 | 1.90 | -0.83 |  |  |  |
| 24-07-2017 | 10.20 | -0.60 |  |  |  |
| 25-07-2017 | 7.60 | -0.12 |  |  |  |
| 26-07-2017 | 1.70 | 0.63 |  |  |  |
| 27-07-2017 | 2.20 | 0.33 |  |  |  |
| 28-07-2017 | 15.30 | -0.62 |  |  |  |
| 29-07-2017 | 3.40 | -0.30 |  |  |  |
| 30-07-2017 | 1.30 | -9.06 |  |  |  |
| 01-08-2017 | 1.30 | -0.23 |  |  |  |
| 03-08-2017 | 1.90 | 0.16 |  |  |  |
| 04-08-2017 | 3.50 | 0.22 |  |  |  |
| 10-08-2017 | 1.50 | -1.06 |  |  |  |
| 14-08-2017 | 0.90 | 0.88 |  |  |  |
| 19-08-2017 | 1.20 | 1.15 |  |  |  |
| 20-08-2017 | 70.30 | -2.68 |  |  |  |
| 21-08-2017 | 21.50 | -2.90 |  |  |  |
| 22-08-2017 | 2.80 | -0.86 |  |  |  |
| 23-08-2017 | 0.20 | 0.90 |  |  |  |
| 24-08-2017 | 1.60 | 0.97 |  |  |  |
